# Safety and Efficacy of Transcatheter Administration of Tissue Plasminogen Activating Factor as Adjuvant Therapy for Intraventricular Hemorrhage

**DOI:** 10.7759/cureus.5785

**Published:** 2019-09-27

**Authors:** Mark Krel, James Brazdzionis, Stacey Podkovik, Dan E Miulli, Margaret Rose Wacker, Yancey Beamer

**Affiliations:** 1 Neurosurgery, Riverside University Health System Medical Center, Moreno Valley, USA; 2 Neurosurgery, Arrowhead Regional Medical Center, Colton, USA

**Keywords:** cerebrovascular accident, cerebral hemorrhage, intraventricular injections, tpa, intraventricular hemorrhage, intracranial hemorrhage

## Abstract

Objective

Stroke is the fifth leading cause of death in the United States and the leading cause of disability. Hemorrhagic stroke has higher risks of mortality and neurologic deficit. Higher still, acute intraventricular hemorrhage (IVH) has mortality between 50% and 80% while complicating subarachnoid hemorrhage in 15% of cases and intracerebral hemorrhage in 40% of cases. We sought to demonstrate that early adjuvant intraventricular recombinant tissue plasminogen activating factor (rt-PA) improved outcomes.

Methods

Retrospective chart review was performed on patients aged 18-95 years with external ventricular drain (EVD) and intraventricular rt-PA for clot evacuation in IVH between 2005 and 2015. In total, 22 patients met the inclusion criteria. Generalized linear modeling was performed with factorial analysis using the Glasgow Coma Score (GCS) on arrival, GCS at EVD placement, EVD day of onset of rt-PA administration, GCS at onset of rt-PA administration, total duration of EVD, necessity of ventriculoperitoneal (VP) shunt, occurrence of ventriculitis, day of ventriculitis, GCS after rt-PA, length of stay (LOS) in the intensive care unit (ICU), and hospital disposition.

Results

Presenting GCS affected LOS significantly. Ventriculitis only significantly affected ICU LOS. GCS after rt-PA only significantly affected discharge GCS. EVD day of rt-PA protocol commencement demonstrated significant effects on EVD duration and cerebrospinal fluid (CSF) diversion requirement. Age affected ICU and hospital LOS.

Conclusion

These findings argue for larger prospective trials of EVD day two rt-PA protocol inception in acute IVH. Reported ventriculitis rates with EVDs are 8.8%, while we demonstrated a rate of 18% without significant effects except in ICU LOS. Transcatheter intraventricular rt-PA is safe and effective as an adjuvant in acute spontaneous intraventricular hemorrhage with the greatest benefit of rt-PA protocol at EVD day two.

## Introduction

Stroke is the fifth leading cause of death in the United States and the leading cause of long-term debility. Each year, approximately 795,000 people suffer a stroke. Hemorrhagic stroke has been shown to have an overall higher risk of mortality in the acute phase with the risk of mortality and permanent neurologic deficit being higher still in patients who presented with acute intraventricular hemorrhage (IVH). If associated with acute intraventricular hemorrhage, the mortality rate reported in the literature is 50 - 80% [1-5]. Several groups have addressed this problem and animal models suggest substantial physiologic and functional benefits associated with early removal of blood clots from ventricular or parenchymal spaces [6,7]. Among the largest and best known of these trials is the Clot Lysis Evacuation of Accelerated Resolution (CLEAR) series of trials. In brief, these trials suggest the feasibility and efficacy of transcatheter administration of recombinant tissue plasminogen activator (rt-PA) for acute intraventricular hemorrhage. They have also demonstrated a concomitant reduction in mortality [8].

Prior investigators have postulated on the mechanism of increased morbidity and worsened outcomes in patients suffering from intraventricular hemorrhage [2,3,6,7,9-15]. IVH complicates subarachnoid hemorrhage in 15% of cases and intracerebral hemorrhage in 40% of cases [1,4,5,8]. If the IVH is large enough to impede cerebrospinal fluid (CSF) outflow, obstructive hydrocephalus will result. In the vast majority of cases, this patient presentation will require CSF diversion [9]. Standard of care is that this diversion occurs emergently by placement of an external ventricular drain (EVD) and if there is insufficient resolution of the clot to return to near-physiologic CSF circulation, the patient will require permanent CSF diversion in the form of ventriculoperitoneal shunt (VPS) or other CSF diversionary shunt placement [3,11,15-19]. As a general rule, it is advisable to avoid permanent implantation of foreign hardware when possible and, therefore, it would be beneficial if a mechanism of avoidance of VPS placement in the face of acute IVH were discovered.

This problem, however, is not limited to IVH in the acute phase. In the subacute and chronic stages of intraventricular hemorrhage, communicating hydrocephalus may develop. This is thought to be caused by fibrosis of the leptomeninges or impaired CSF absorption due to fibrosis of the arachnoid villi [1,2,7,11,12]. Blood and blood derivatives are well-known pro-inflammatory agents in neurologic disease. This inherent proinflammatory nature of the hemorrhage combined with biologic tissue's innate response to physical injury is likely the mechanism for the fibrosis mentioned. In the acute phase, IVH is almost always associated with an acutely elevated intracranial pressure (ICP). The alteration of consciousness associated with acute IVH, then, is likely due to intracranial hypertension induced ischemic encephalopathy. As ICP increases, cerebral perfusion pressure (CPP) decreases, and this disproportionation contributes to ischemia. Additionally, direct compression of the rostral brain stem and thalamus from local mass effect may contribute to pathology [10,12,14,19-21].

Factors associated with poor prognosis in IVH include ventricular dilatation, IVH volume, and increased ICP. It is intuitive that all three negative prognostic factors would co-occur in the setting of large IVH as the clot will tend to obstruct normal CSF outflow causing obstructive hydrocephalus, thereby dilating the ventricular system and increasing ICP. In general, the standard of care treatment for acute IVH centers primarily on the management of the increased ICP. EVD placement is the standard of care for clinical or radiographic manifestations of acute obstructive hydrocephalus. It is worth noting that not all patients who present with acute obstructive IVH will have increased ICP and not all patients in whom ICP is ultimately controlled will have an improvement in neurologic deficit [2,6,11,12,14,15,17,18]. Coplin et al. demonstrated that poor outcome in patients with large IVH who were untreated occurred in 66.7% of patients while those who were treated with transcatheter thrombolytics (urokinase in their study) experienced poor outcomes in 31.8% of cases [22].

There is compelling evidence to suggest that clot evacuation improves patient outcomes and that transcatheter intraventricular thrombolytics facilitate this process. To date, however, we can find no strong evidence that suggests a causal relationship between the timing of commencement of thrombolytic therapy and specific outcome measures in patients with acute IVH [6-8,10,11,13,16-26]. We aim to demonstrate that there exists an optimal timing at which rt-PA protocol can be initiated to maximize patient outcomes. The variables used as measures of outcome were duration of EVD, whether the patient developed ventriculitis, whether a shunt was necessary, the final Glasgow coma scale (GCS) (after rt-PA protocol, or, if different, at disposition), length of stay (LOS) in the intensive care unit (ICU) and hospital, and disposition. We sought to demonstrate that patients who were begun on transcatheter intraventricular administration of rt-PA earlier had more favorable outcomes.

## Materials and methods

A retrospective chart review was performed on all patients aged 18-95 who had an external ventricular drain placed and received intraventricular administration of recombinant tissue plasminogen activating factor to facilitate clot evacuation in intraventricular hemorrhage between 2005 and 2015. Patients whose families elected to withdraw care before removal of EVD or whose ventricular hemorrhage was associated with hemorrhagic ventricular mass lesions were excluded from participation in the study. Patients were selected from the historical census searching for International Statistical Classification of Diseases and Related Health Problems (ICD) 10 code I61.9, ICD 9 code 431 and keywords in presenting problems including intraventricular hemorrhage, casted ventricles, ventricular hemorrhage, and ventricular extension.

rt-PA was administered using a uniform protocol for all patients who receive transcatheter intraventricular rt-PA in our practice; a dosage of 2 milligrams in 2 milliliters instilled into the ventricular catheter. Patients were eligible for inclusion in this study if they experienced an intraventricular hemorrhage resulting in hydrocephalus, which was treated with an external ventricular drain and subsequent transcatheter intraventricular rt-PA. Patients were excluded from the study if any of the holes from the external ventricular drain catheter were located in the brain parenchyma. Systolic blood pressure (SBP) was maintained below 140 mmHg at all times. It was maintained below 130 mmHg one hour before, during, and one hour after rt-PA administration. At times rt-PA administration was held until SBP was controlled, delaying the day of medication admission. Then, every 12 hours, the rt-PA was infiltrated into the ventricle via the EVD catheter, and the system was clamped for thirty minutes. Once thirty minutes had elapsed, the drainage system was reopened. A non-contrast enhanced computed tomography scan (NCCT) of the head was performed after eight doses of rt-PA. If there was no mass effect, there was evidence of the ventricular flow of CSF, and > 50% reduction in IVH volume then the EVD was trialed on a wean. In a typical case wherein the patient was easily amenable to weaning, on a 24-hour schedule, the EVD was raised from six mmHg to 10 mmHg, then to 15 mmHg, then 20 mmHg. Twenty-four hours after having been raised to 20 mmHg and tolerated by the patient, the EVD would be clamped for 24 hours. During this weaning process, the rt-PA dosing protocol would be continued as described. After 24 hours of clamping, the patient would again receive an NCCT of the head to ensure that there was no ventricular dilatation. The EVD would then be removed in a typical way after at least six hours had elapsed since the last administration of rt-PA.

Data were collected from the electronic medical record at our institution. The following data points were collected: Glasgow Coma Scale on arrival, GCS at time of placement of external ventricular drain (if different than presenting GCS), GCS at onset of rt-PA administration protocol, days since EVD placement at day of onset of rt-PA administration protocol, whether hydrocephalus was present on admission, whether the EVD was positioned ipsilateral or contralateral to the largest intraventricular clot, total duration of EVD, whether a CSF shunting procedure was ultimately necessary, whether the patient suffered ventriculitis (and if so, the day of ventriculitis relative to the day of EVD and to the day of rt-PA), GCS at conclusion of rt-PA protocol, length of stay in the intensive care unit, total length of stay in the hospital, and post-hospital disposition as a proxy for patient condition on discharge. Of these, outcome variables included the duration of EVD, whether the patient developed ventriculitis, whether a shunt was necessary, the final GCS (after rt-PA protocol, or, if different, at disposition), length of stay in ICU and hospital, and disposition.

For analysis purposes, predictor variables, therefore, included GCS on arrival and GCS at EVD placement, GCS at rt-PA protocol onset, the occurrence of ventriculitis, GCS after rt-PA protocol, EVD day of rt-PA protocol onset, and patient age. Of note, ventriculitis occurrence was used both as a predictor and outcome variable as its occurrence seemed likely to affect the patient's disease course and there was a presumed association between intraventricular administration of medication and increased rates of ventriculitis. Data were analyzed using a Generalized Linear Model accounting for omnibus and main effects. Factorial analysis could not be performed due to the large difference in degrees of freedom between the different predictor variables and is therefore omitted moving forward. Analysis was completed using IBM SPSS version 23.0 (IBM Corporation, Armonk, North Castle, NY). This study was approved by the institutional review board at Arrowhead Regional Medical Center, Colton, CA, USA. Due to the nature of the data collection, which utilized a chart review, the review board waived the requirement for formal informed consent.

## Results

A total of twenty-two patients met all inclusion criteria for this study. The ages of patients included in this study ranged from 18 to 79 years of age with a mean age of 53.82 years. GCS on arrival ranged from four to 14 with mean intake GCS of eight. GCS at the onset of rt-PA protocol ranged from three to 14 with mean 9.14. GCS after completion of rt-PA protocol ranged from eight to 15 with mean 11.86. The patients at GCS of < eight remained intubated and/or with surgical airway until the GCS improved and the patient was able to be removed from the ventilator. The patient was either transferred to a long-term acute care facility (LTAC) or the patient expired. GCS at discharge ranged from eight to 15 with a mean of 12.32.

Four patients enrolled in the study developed ventriculitis. Of those, one developed ventriculitis prior to commencement of rt-PA protocol. EVD day of ventriculitis ranged from six to 22 with mean 11. The day the patient developed ventriculitis after rt-PA administration ranged from -2 (the patient who developed ventriculitis prior to rt-PA administration) to nine with a mean of five. Only two patients required permanent CSF diversion. Length of stay in ICU ranged from five to 160 days with a mean of 21.82 days. The total duration of hospitalization ranged from nine to 224 days with mean 44.14 days. See Table 1 for a summary of these descriptive statistics.

**Table 1:** Summary descriptive statistics of patients enrolled in this study Abbreviations: Glasgow Coma Scale (GCS), Tissue Plasminogen Activator (tPA), External Ventricular Drain (EVD), Length of Stay (LOS), Intensive Care Unit (ICU)

	N	Minimum	Maximum	Mean	Standard Deviation
Age	22	18	79	53.82	11.562
GCS on arrival	22	4	14	8.00	3.309
GCS at tPA	22	3	14	9.14	2.696
GCS p tPA	22	8	15	11.86	2.513
GCS at Discharge	22	8	15	12.32	2.418
EVD length	22	5	39	13.32	8.796
EVD Day of tPA	22	1	24	3.23	4.720
tPA Day of ventriculitis	22	-2	9	5.13	3.716
EVD Day of ventriculitis	22	6	22	11.33	5.487
LOS ICU	22	5	160	21.82	32.238
LOS Hospital	22	9	224	44.14	55.953

**Figure 1:**
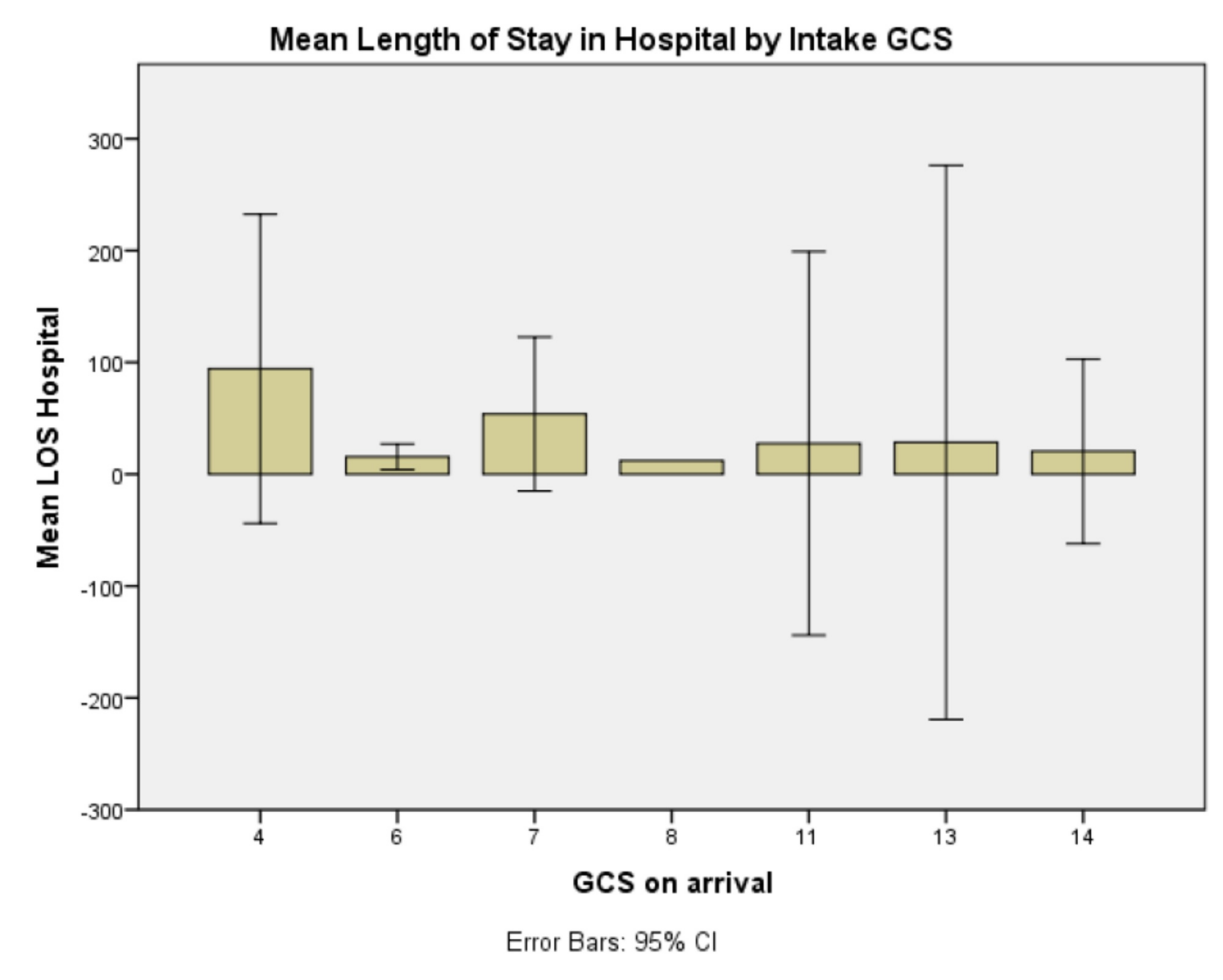
Mean length of stay in hospital by intake Glasgow Coma Scale (GCS) This graph demonstrates a bimodal distribution of length of stay in hospital with longest stays noted at Glasgow Coma Scale four and seven at intake and shortest stay noted at GCS eight at intake. Abbreviations: Glasgow Coma Scale (GCS), Length of Stay (LOS)

**Figure 2:**
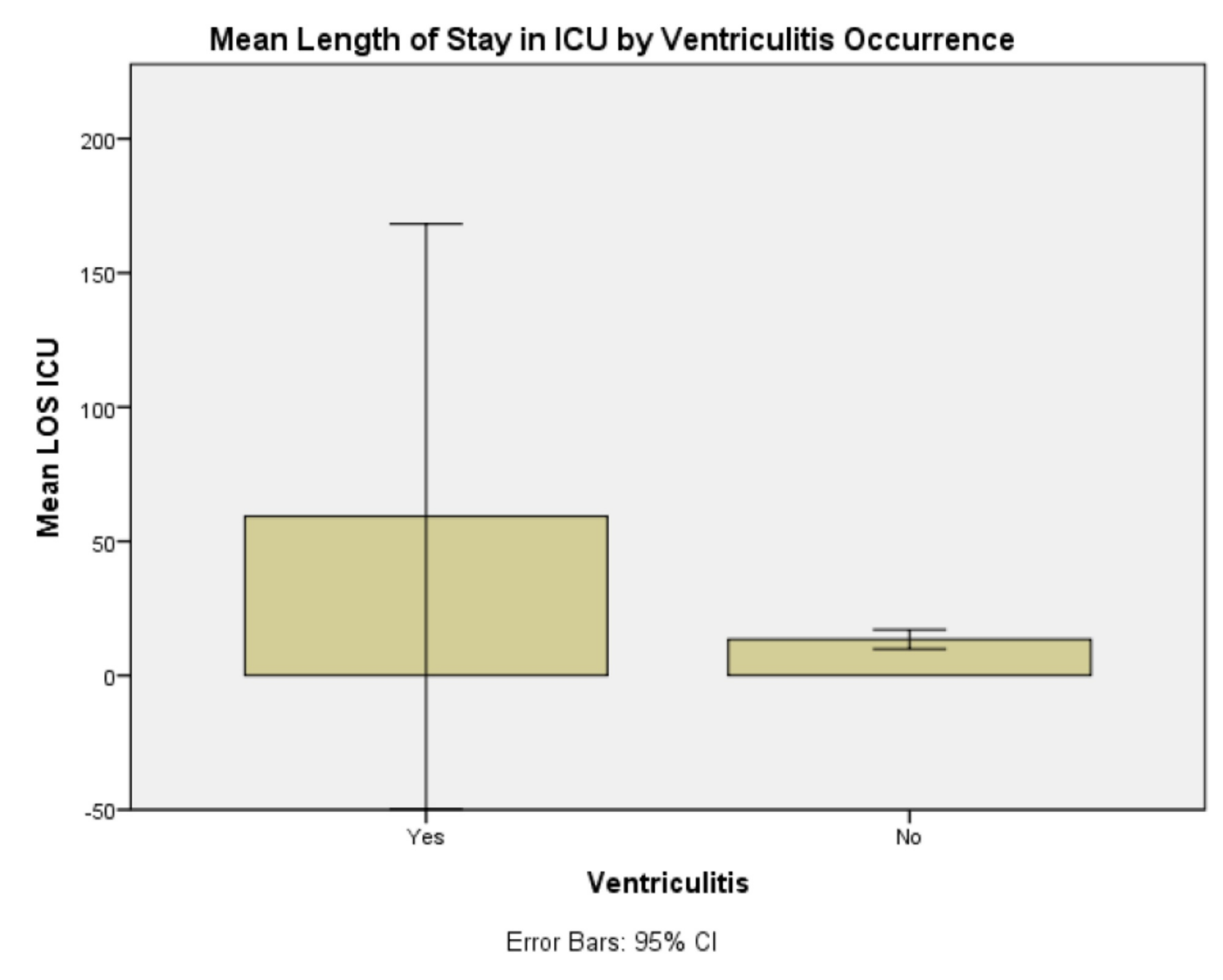
Mean length of stay in the Intensive Care Unit (ICU) by ventriculitis occurrence This graph demonstrates that mean length of stay in the Intensive Care Unit is very increased in the event of occurrence of ventriculitis. Abbreviations: Intensive Care Unit (ICU), Length of Stay (LOS)

The test of omnibus effects of the relationship was significant, F(9.68) = 84671.943, p < 0.0005. The omnibus test being significant means that some or all features of the comparison are statistically valid and statistically significant. Presenting GCS had no significant effect on EVD duration, need for CSF diversion, LOS in ICU, GCS at discharge, or patient disposition. It did, however, have a significant effect on LOS in hospital, F(7,21) = 4155.828, p < 0.0005 (Figure 1). GCS at time of rt-PA protocol commencement had no significant effect on EVD duration, need for CSF diversion, LOS in ICU, GCS at discharge, or patient disposition. Occurrence of ventriculitis demonstrated a significant effect only on length of stay in ICU, F(1,21) = 9.148, p = 0.007 (Figure 2). GCS after completion of rt-PA protocol only demonstrated a significant effect on GCS at time of discharge, F(6,21) = 13.943, p < 0.0005.

**Figure 3:**
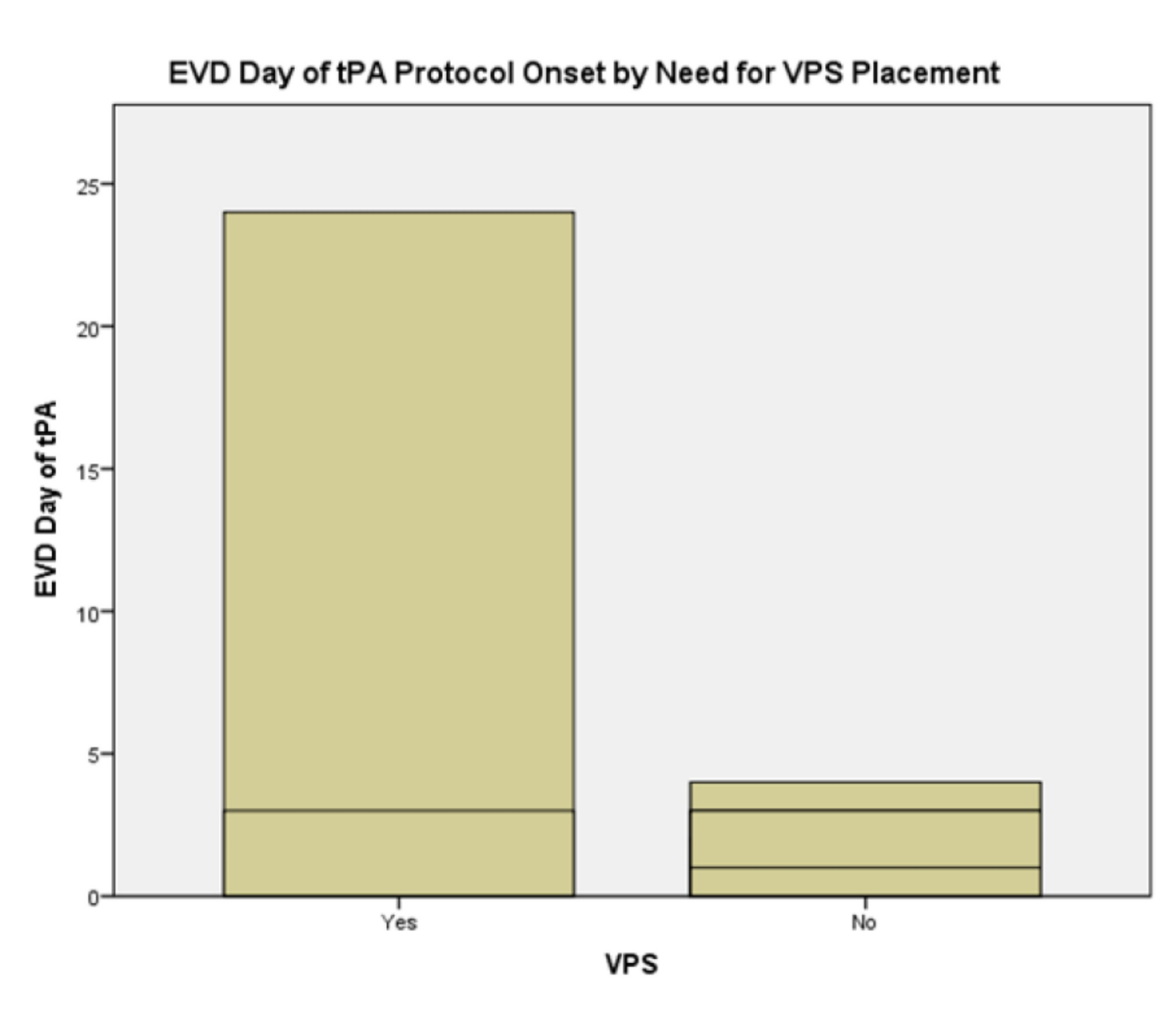
External Ventricular Drain (EVD) Day of Recombinant Tissue Plasminogen Activator (rt-PA) protocol onset by need for cerebrospinal fluid diversion Notably, patients who required ultimate Ventriculoperitoneal Shunt placement were begun on Recombinant Tissue Plasminogen Activator protocol later in their hospital course than were the patients who did not require VPS placement. Abbreviations: External Ventricular Drain (EVD), tPA (Tissue Plasminogen Activator), Ventriculoperitoneal Shunt (VPS)

**Figure 4:**
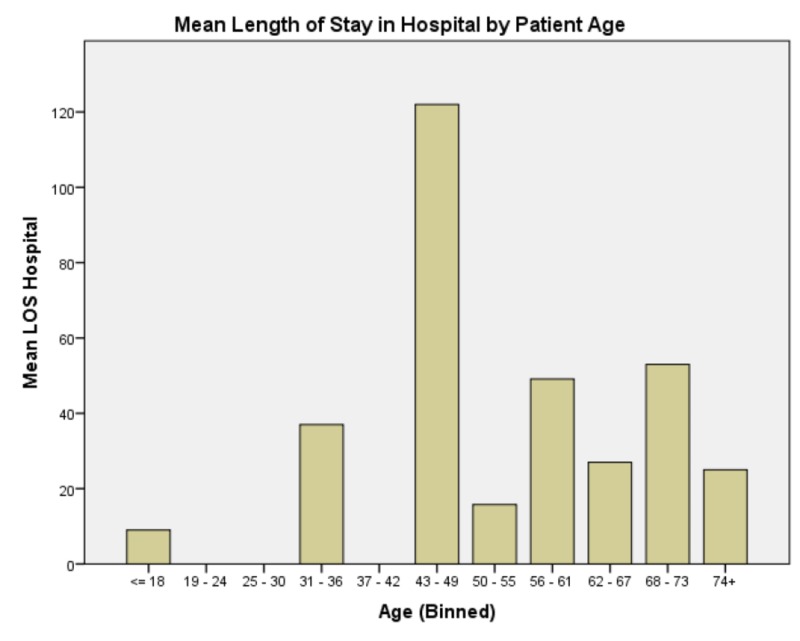
Mean length of stay in the Hospital by patient age Generally, there is a trend that older patients have longer hospital stays. The clear exception to this, however, is the 43-49 age bin that had the longest overall mean hospital stay. This is multifactorial and not strictly attributable to medical problems but also is accounted for by social and economic factors. Abbreviations: Length of Stay (LOS)

**Figure 5:**
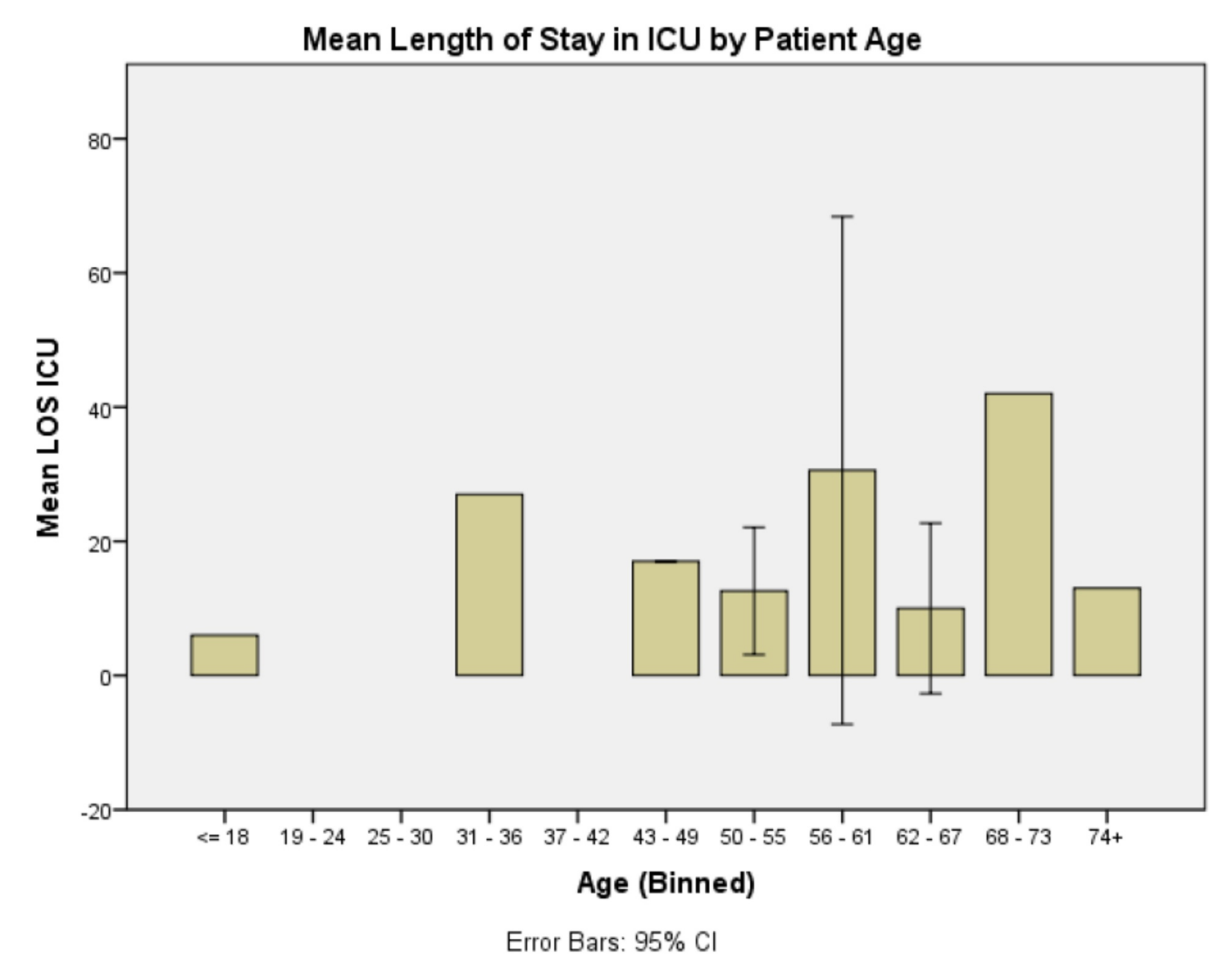
Mean length of stay in the Intensive Care Unit (ICU) by patient age Generally, there is a trend that older patients have longer intensive care unit (ICU) stays, and this trend was found to be statistically significant. Abbreviations: Abbreviations: Length of Stay (LOS), Intensive Care Unit (ICU)

EVD day of rt-PA protocol commencement demonstrated significant effects on total duration of EVD and need for CSF diversion; F(4,21) = 5.026, p = 0.007, and F(4,21) = 5.409, p = 0.005 respectively. No patients who received rt-PA protocol at EVD day two or sooner required ventriculoperitoneal (VP) shunt placement; 20% of patients who received rt-PA at EVD day three required VP shunt placement; and all patients who received rt-PA protocol commencing at EVD day four or later required VPS placement (see Figure 3). Patient age had no predictive value for EVD duration, need for CSF diversion, GCS at discharge or patient disposition. It did, however, significantly affect length of stay both in the ICU and in the hospital overall; F(14,21) = 30.472, p < 0.005, and F(13,20) = 13.039, p = 0.001 respectively (Figures 4, 5). In summary, presenting GCS significantly predicts total LOS in hospital but not in ICU with a bimodal distribution with GCS of four and seven at intake portending the longest hospital stays and an intake GCS of eight, the shortest. GCS at onset of rt-PA protocol did not significantly affect any of our outcome variables. Patients whose GCS was improved after rt-PA protocol had similar improved GCS at time of hospital discharge. The occurrence of ventriculitis predicts longer ICU stays but not necessarily overall LOS in hospital and, of note, does not significantly lengthen duration of EVD or predict poor outcome. EVD day of rt-PA protocol commencement significantly affects total EVD duration and ultimate need for CSF diversion. Patients who received rt-PA protocol sooner had shorter EVD courses. Patient age significantly affects the length of stay in ICU and overall hospital stay (see Table 2). Lastly, patient age predicted both ICU and Hospital LOS. Patients aged 43 - 49 had the longest hospital stays and patients aged 68 - 73 had the longest ICU stays. Importantly, presence of hydrocephalus at admission and sidedness of the EVD had no significant effect on any of the dependent variables listed in Methods.

**Table 2:** Summary of significant effects This table summarizes all the analyzed variables that had significant effects and the variables that they had significant effects on along with the relevant p-values. Abbreviations: Glasgow Coma Scale (GCS), Recombinant Tissue Plasminogen Activator (rTPA), External Ventricular Drain (EVD), Length of Stay (LOS), Intensive Care Unit (ICU), Ventriculoperitoneal (VP)

Factor	Effect	p-value
Presenting GCS	Hospital LOS	< 0.0005
GCS after rtPA protocol	GCS at discharge	< 0.0005
Ventriculitis	ICU LOS	0.007
EVD day at time of rtPA protocol onset	Total EVD duration	0.007
EVD day at time of rtPA protocol onset	Need for VP Shunt	0.005
Age	ICU LOS	< 0.0005
Age	Hospital LOS	0.001

## Discussion

Recombinant tissue plasminogen activating factor has long been shown to be safe and effective in declotting vascular catheters that become clotted with hematologic material. It stood to reason that the same mechanism should provide for increased efficiency of clot resolution in body spaces to which surgical access is gained. Of particular interest in this study are clots consequent to intraventricular hemorrhage in patients who had external ventricular drains placed for intracranial pressure monitoring and management and clot evacuation. Several studies have examined the safety and efficacy of this treatment paradigm in both animal models and human trials. These have included various disease states such as spontaneous hypertensive hemorrhage, trauma, and aneurysmal hemorrhage [6,8,20-28,10-13,15-17,19]. As all of these had been previously demonstrated to be useful applications for rt-PA transcatheter implementation, no distinction was made between these etiologies for the purpose of this analysis.

Several of the results presented in this study are intuitive and not surprising. For example, GCS at cessation of the rt-PA protocol was tightly and significantly linked with discharge GCS (see Table 2). Barring catastrophic events such as re-bleeding (which did not occur in our sample), superinfection, iatrogenic injury, or respiratory failure, it stands to reason that patients who are doing clinically better at the termination of medicinal therapy will also be relatively clinically better at time of hospital discharge. Others were more clinically meaningful - there is no statistically significant relationship between the presence of hydrocephalus at admission and any of our outcome measures. Of note, all patients included in this study, except one, presented with some degree of acute obstructive hydrocephalus and this, in part, is the condition the EVDs were placed to treat. Of note, as well, one patient in our sample was GCS 14 at presentation but received an EVD during his course because of acute deterioration in clinical status and GCS. It is reasonable, therefore, to infer that with early intervention in a picture of acute obstructive hydrocephalus, EVD is effective management and, furthermore, that transcatheter application of rt-PA facilitates and speeds clot lysis, removing the obstruction, and expediting return to physiologic CSF circulation.

EVD laterality had no significant effect on total EVD duration, need for VPS placement, LOS in ICU, LOS overall in the hospital, GCS at discharge, or patient disposition. This is in agreement with the work of Jaffe and colleagues whose work demonstrated laterality, in and of itself, had no bearing on intraventricular clot evacuation, but that the catheter being placed within the largest volume of the clot does increase the speed of clot evacuation [18]. Speed of clot evacuation has been demonstrated widely to be significant at a physiologic level (i.e. to return biochemical and biophysical processes of cerebral metabolism and CSF circulation to pre-incident baseline) and has been postulated to be of clinical importance, however, a thorough discussion of this point is outside the scope of the present study. Of note, all patients in this sample had a complete or near-complete resolution of intraventricular blood visible by conventional NCCT scan of the head. EVD duration, however, is not strictly dependent on computed tomography (CT) evidence of clot resolution. General EVD weaning protocol involves successive challenges to the CSF circulatory system by progressive raising the height of the EVD and close monitoring of neurologic exam and biometric parameters [9,11,15,17,18]. Clot resolution, inherently, is an important part of reestablishing physiologic CSF circulation and will, in this way, assist in weaning the EVD. It is not, however, the sole factor responsible for the ability to wean. For this reason, we elected to use overall EVD duration as a marker for clinical progress rather than strictly CT resolution of intraventricular hemorrhage as, often, this was insufficient to wean the EVD and, in some cases, patients with CT resolution of IVH still required VPS placement. Other authors have proposed a pathologic mechanism for this involving fibrosis of the basal leptomeninges or impaired CSF absorption due to fibrosis of arachnoid villi leading to communicating hydrocephalus even in the face of resolution of the presenting acute obstructive hydrocephalus. In our study rt-PA continues to be given as the EVD is weaned possibly using an increasing pressure head to push rt-PA into the basal leptomeninges or arachnoid villi decreasing the rate of fibrosis. The overall rate of need for VP shunt placement in our sample of patients, all of whom underwent the rt-PA protocol as outlined above, was 8%. This is much lower than the rate of shunt dependence by EVD management alone reported in the literature that ranges from 55-65% [1,5,11,15,17,18].

As could be reasonably expected, patients who present with low GCS tend to have longer hospital stays. They do not necessarily have longer ICU stays. This may be due to the fact that patients who present with low GCS also tend to have more severe medical comorbidities that require more protracted hospital courses. However, the neurosurgical issues caused by the presence of anatomic pathology - in this case, acute intraventricular hemorrhage - are resolved and these patients do not require longer ICU courses. Patients who present at GCS of eight seemed to have the shortest length of stay in both ICU and hospital. Perhaps this is attributable to acute encephalopathy that is reversible while patients who have lower GCS (e.g. GCS four) have more difficult to overcome or more extensive injury as the injuries to the brain grave enough to cause a GCS three or four, generally affect “lower” brain functions, particularly those related to functions of living. Review of existing literature on length of stay with intracranial hemorrhage shows that patients who present with GCS 10 or better have shorter stays and seven or worse have significantly longer stays (average 12 for > 10, 31 for < seven) (in the setting of intracranial hemorrhage). When IVH is comorbid, that increases the length of stay but not significantly. Furthermore, the average length of stay ranges from nine to 22 days. Many of the patients in this study had more protracted stays than this reported average, however, this may be attributable to the patient population our safety-net hospital serves and the concomitant difficulty in safe disposition after inpatient hospitalization inherent to this population [29].

Supporting the notion that people with already diminished functional reserve have more protracted disease courses, older patients tended to have longer ICU and hospital stays overall. Of note, however, patients who fell in the 43 - 49 age group had the longest hospital stay by a wide margin without a proportionally longer ICU stay than other groups. These patients had slightly longer-than-expected ICU stays but dramatically longer hospital stays (see Figures 4, 5). This is attributable to a variety of socioeconomic issues rather than frank medical issues. These patients, in general, were more likely to be polysubstance abusers who had either never been under the care of a primary physician, were medically noncompliant, or had otherwise difficult to control comorbidities especially hypertension and diabetes contributing to microvascular pathology that can underlay intracranial hemorrhage. Furthermore, these patients tended to be either sole- or primary providers in their respective social dynamics and, therefore, had fewer options for safe discharge and required extended hospitalization to seek placement including ancillary tasks to this goal including acquiring funding for post-acute placement.

Anecdotally, several neurosurgeons have trepidation with initiating transcatheter medication administration that requires multiple, repeated pushes of fluid directly into the ventricular space due to increased risk of ventriculitis. Previous data indicate an overall rate of ventriculitis with EVD catheters of 8.8% while in the present study, the rate of ventriculitis in this study was 18% [30]. Certainly, this is a notable and clinically important rate increase. Of equal importance, however, is that there was no statistically significant effect found in any of the measured outcome variables except length of ICU stay. ICU stay tends to be the most costly portion of a patient’s hospitalization - particularly with procedural intervention and neurosurgical management. Care should be taken to minimize the risks of ventriculitis and future work will define strict protocols for optimized sterility of administration. The fear of ventriculitis, however, should not preclude implementation of transcatheter intraventricular administration of rt-PA to resolve acute intraventricular hemorrhage.

Perhaps most meaningfully, the duration of time elapsed between the placement of the EVD catheter and onset of the rt-PA protocol had significant effects on both duration of EVD and the ultimate need for permanent CSF diversion in the form of a ventriculoperitoneal or other shunt (see Figure 3, Table 2). Generally, when rt-PA protocol is started later in the EVD course, patients were more likely to require VPS placement and had a longer duration of EVD. The overall rate of need for VPS placement in our sample of patients, all of whom underwent the rt-PA protocol as outlined above, was 8%. This is much lower than the rate of shunt dependence by EVD management alone reported in the literature that ranges from 55-65% [1,5,11,15,17,18]. Interestingly, the distribution of EVD duration is U-shaped with the nadir occurring when rt-PA protocol is begun at EVD day two (24-48 hours after EVD placement). Mean EVD duration when rt-PA protocol is commenced within 24 hours of EVD placement is 14.5 days compared with 9.1 days for patients whose rt-PA protocol commenced on EVD day two. For patients whose rt-PA protocol was commenced on EVD day three, the mean EVD duration returned to 14 days. A concise statement on the etiology subtending this finding would require a more granular investigation into the comorbidities and presenting problems of the patients in the respective groups and will be left for future investigation with a larger sample size and would benefit from a prospective design approach. No patients who received rt-PA protocol at EVD day two or sooner required VP shunt placement; 20% of patients who received rt-PA at EVD day three required VPS placement; and all patients who received rt-PA protocol commencing at EVD day four or later required VPS placement. The reason most patients received rt-PA later than day two were due to inability to control systolic blood pressure to within treatment parameters. It is unclear as to whether starting rt-PA earlier in patients with higher blood pressures would affect outcomes as defined in the Methods section.

## Conclusions

Although limited by small sample size, the calculated probability of a type I error in all conditions that proved to be significant was quite small. While this is insufficient for Level Ia recommendation to clinical practice, it argues strongly in favor of larger prospective trials of rt-PA protocol inception at EVD day two for patients presenting with acute intraventricular hemorrhage. Furthermore, it is intuitive that these symptomatic patients will present, albeit at varying stages of evolution, with acute obstructive hydrocephalus that will be addressed by standard of care emergent EVD placement. In conclusion, we argue that rt-PA is a safe and effective adjuvant therapy for transcatheter intraventricular administration in the setting of acute intraventricular hemorrhage from all causes except that caused by hemorrhagic periventricular or intraventricular mass lesions. ICU and Hospital LOS, GCS at discharge, EVD duration, and need for VP shunt are significantly improved with early rt-PA intraventricular administration. Moreover, we suggest that rt-PA protocol offers the greatest benefit to patient care and outcomes when it is begun at EVD day two to decrease the overall rate of need for VPS placement to 8% which is much lower than the rate of shunt dependence 55-65% by EVD management alone reported in literature.
